# Improved pathology reporting in NAFLD/NASH for clinical trials

**DOI:** 10.1136/jclinpath-2021-207967

**Published:** 2021-11-09

**Authors:** Caitlin Rose Langford, Marc H Goldinger, Darren Treanor, Clare McGenity, Jonathan R Dillman, Daniela S Allende, Robert Goldin, Elizabeth M Brunt, Kurt Zatloukal, Helmut Denk, Kenneth A Fleming

**Affiliations:** 1 Perspectum Ltd, Oxford, UK; 2 Pathology, University of Leeds, Leeds, UK; 3 Department of Histopathology, Leeds Teaching Hospitals NHS Trust, Leeds, UK; 4 Department of Radiology, Cincinnati Children's Hospital Medical Center, Cincinnati, Ohio, USA; 5 Department of Radiology, University of Cincinnati College of Medicine, Cincinnati, Ohio, USA; 6 Department of Anatomical Pathology, Cleveland Clinic, Cleveland, Ohio, USA; 7 Section for Pathology, Imperial College, London, UK; 8 Department of Pathology and Immunology, Washington University School of Medicine in Saint Louis, St Louis, Missouri, USA; 9 Diagnostic and Research Institute of Pathology, Medical University of Graz, Graz, Austria; 10 Green Templeton College, University of Oxford, Oxford, UK

**Keywords:** liver diseases, gastrointestinal diseases, liver, safety, research report

## Introduction

Non-alcoholic fatty liver disease (NAFLD), a disorder characterised by pathological accumulation of non-visible free fatty acids and visible triglyceride in hepatocytes, is on the rise globally in both adult and paediatric populations.^1^ Evidence suggests that 20%–50% of the European Union and US populations exhibit features of NAFLD,^2^ driven by higher rates of obesity, insulin resistance and type 2 diabetes, and metabolic syndrome.^3^ Additionally, recognition of a growing number of patients with ‘lean NAFLD’ who are not obese, but have high levels of visceral fat, diets high in fats and carbohydrates, or who have genetic risk factors, has increased.^4^ Of patients with NAFLD, 6%–55% will have histological signs of non-alcoholic steatohepatitis (NASH), which if left unmanaged can lead to cirrhosis and potentially hepatocellular carcinoma.^5^

NAFLD has surpassed viral hepatitis as the leading cause of chronic liver disease worldwide.^6^ Estimates suggest that by 2030 NAFLD will overtake hepatitis C as the primary cause of liver failure requiring transplantation and that the number of NAFLD-related deaths will increase by 178%.^7^ Annual spending related to NAFLD care is estimated to rise exponentially from $103 billion to $1.005 trillion in the USA and from €35 billion to €334 billion in Europe between 2016 and 2025.^8^ While bariatric surgery and/or weight loss can be effective, they present their own challenges in delivery. There are presently no approved drugs on the market; hence, the number of clinical trials has grown by approximately 60% over the last 10 years.

As both the Food and Drug Administration and the European Medicines Agency require liver biopsy for clinical trials as the ‘gold standard’, diagnosis and monitoring rely on pathological assessment of a liver biopsy.^9–11^ Most trials focus only on the numerically reported values of the semiquantitative assessment of four cardinal features of NAFLD/NASH—steatosis, inflammation, hepatocellular ballooning and fibrosis—as set out by the Pathology Committee of the NASH Clinical Research Network.^12^ However, there are a number of potential problems (see next section), and a recent study argued that poor reliability of liver biopsy evaluation in NAFLD ‘allows improper entry, misclassification, and diminishes treatment effect’.^13^ Two follow-up editorials from pathologists discussed this further.^14 15^

## Current pathology reporting in NAFLD/NASH clinical trials

As mentioned above, the reliance on focused scoring systems alone has several potential weaknesses. First, there is no requirement to comment on sample adequacy unless the sponsor and/or the pathologists involved specified this in the protocol. As liver biopsy specimens typically represent approximately 1/50 000th of the entire liver volume, inadequate biopsy length can greatly affect the quality of assessment.^16 17^ It is generally recommended that all medical liver biopsies should be at least 25 mm long and of sufficient width (~1.6 mm) to include at least 10 portal tracts.^18 19^ Therefore, comments on sample adequacy should be required.

Second, trials typically require only H&E and one connective tissue stain,^9 12^ deviating from what medical societies, including the UK Royal College of Pathologists (RCPath) and the American Association for the Study of Liver Diseases, have outlined as robust practice in clinical care.^20 21^ Up to seven stains are recommended as certain features cannot be identified without a particular stain (eg, Shikata’s orcein for long-standing cholestasis and a Perls’ stain for iron), any of which could be highly relevant to assessing a patient’s response in a trial. Furthermore, as biopsies are often processed and stained at multiple sites, the quality of processing and/or staining should be noted due to difficulties in assessing disease features if these are suboptimal. Requiring comments on the quality and utility of staining would address these issues.

Third, focusing solely on four features alone and ignoring others can cause major problems. This narrow focus was implicated in the placing of a clinical hold on a recent drug trial in which biopsies were subsequently found to have features atypical for NASH. It was unclear whether these subjects had newly developed liver injury or had pre-existing changes and should have been excluded from the trial in the first place.^13 15 22^ Another major reason for noting such abnormalities is that response to the trial drug may be influenced by their presence and therefore may be relevant to the trial’s outcome. Within the current system, aside from the possibility that some features will not be recognised without the appropriate stain, if there are any additional pathological features, the expectation is that these will be noted in the ‘comment’ section. However, in the absence of any ‘comment’, it is unclear if there are no such additional findings, or that there are but were not detected as a result of the pathologist only examining for NAFLD Activity Score (NAS) components, or if the pathologist had observed them but assumed that trial sponsors do not require the information.

Fourth, as histopathology is subjective, interobserver and intraobserver variations in assessing features are inevitable.^13 23^ The fundamental cause of this variability is that definitions of NASH features are not sufficiently specific for different pathologists to identify them reliably. Requiring much more precise and agreed definitions for both recognition and quantification of the features will reduce the degree of variation. As an illustration of the potential, ensuring pathologists within a single trial agree on definitions of features before the start of a trial and/or assess biopsies simultaneously alleviates some of the issues.^24^

## Synoptic reporting: comprehensive, structured reporting

To deal with and potentially overcome the above concerns, we propose the most appropriate method for NAFLD trial pathology reporting is the adoption of synoptic reporting, which ensures all features salient to the diagnosis and monitoring of liver disease/injury and potential treatment effect/resolution are reported.

Synoptic reporting has been adopted in radiology and cancer pathology clinical practice to overcome two main inherent shortfalls of narrative reporting—failure to report all the relevant features and failure to provide a clear final message—resulting in misinterpretation or misreporting of disease features.^25 26^ Typically, synoptic reports offer three levels of organisation and standardisation: a structured format with paragraphs and subheadings; consistent organisation of subheadings, ensuring all required features are described in a logical order; and standardised language and terminology, which enhances the accessibility of reports to non-specialists and reduces ambiguity.^27^ A key component is the required minimum set of features (data set or checklist) which are judged to be crucial to the accurate assessment of the patient and as such must be addressed in the report.

The implementation of synoptic reporting in clinical practice has been supported by bodies including the RCPath, the Association of Directors of Anatomic and Surgical Pathology, and the College of American Pathologists.^28^ The use of such mandatory reporting parameters has been shown to improve reporting accuracy and completeness across a range of subspecialties, with more complex studies benefiting most.^29–32^

## Implementation of synoptic reporting in NAFLD/NASH trial pathology

We recognise there are several barriers to adoption in NAFLD trial pathology.

First, a key factor is the choice of which features to include/not include in the standard report: the minimum data set. Two factors are required: good/acceptable reproducibility through precise agreed definitions and clinical relevance through correlation of clinical outcome with each feature. There is no point including a feature which is highly clinically relevant but poorly reproducible; conversely there is no value in including a highly reproducible feature of no clinical relevance. Agreement on what constitutes the minimum data set and which features should be included is a major future task and broader than the argument made here for use of synoptic reporting. However, recommendations issued by bodies including the RCPath could form the basis on which synoptic reporting templates are built; online supplemental appendix 1 shows a potential example of a putative data set. It should be noted that using such a data set does not prevent scoring of the current four cardinal features.

10.1136/jclinpath-2021-207967.supp1 Supplementary data

Second, as the flexibility offered by narrative reporting is preferred by some pathologists—it facilitates communications of nuanced diagnoses or microscopic findings—it is important that the adoption of synoptic reports does not diminish a pathologist’s ability to flag such findings. Accordingly, the design of the report must allow for this by including free text boxes for relevant comments.

Lastly, but not unimportantly, when considering a trial sponsor’s adoption of synoptic reporting in NASH clinical trials, the related increased costs will prove some additional expense. While the burden of increased pathology costs may initially appear high, the financial impact of suboptimal reporting is greater.

## Conclusions

It may be that the time has come to accept that reporting of liver biopsies from individuals with NAFLD who are entered into clinical trials can and should be improved. Although unquestionably invaluable, use of NAS as the only pathological endpoint in trials results in an incomplete assessment of liver disease features, both preintervention and postintervention, which in turn can undermine the outcome of the trial.^13^ To address this, we therefore propose the development, testing and adoption of a more comprehensive, structured reporting style—known as synoptic reporting—for use in clinical trials.
