# Supplementary material for: Improved pathology reporting in NAFLD/NASH for clinical trials
Source: J Clin Pathol. 2021 Nov 9;75(2):73–5. doi: 10.1136/jclinpath-2021-207967 (PMC8788244; doi:10.1136/jclinpath-2021-207967)
Supplement: Supplementary data [file jclinpath-2021-207967supp001.pdf]

Appendix 1 Example dataset for liver biopsy reporting in NASH clinical trials

**Sample adequacy**	
Length	x mm
Number of portal tracts	x
Staining quality: H&E	Acceptable/poor
Staining quality: connective tissue (Masson's trichrome or picrosirius red)	Acceptable/poor
Staining quality: reticulin	Acceptable/poor
Staining quality: PASD	Acceptable/poor
Staining quality: PAS	Acceptable/poor
Staining quality: copper stain (orcein or rhodamine)	Acceptable/poor
Staining quality: Perls' iron	Acceptable/poor
**NAS**	
Steatosis	0, 1, 2, 3
Lobular inflammation	0, 1, 2, 3
Ballooning	0, 1, 2
Total NAS	
**Fibrosis**	
CRN Stage	0, 1a, 1b, 1c, 2, 3, 4
Signs of regression	Y/N
	If Y: thin septa; hepatocytes growing into septa or vessel walls, other
**Portal inflammation**	
Grade	Mild, moderate, severe
Cell types	
Diffuse or focal	Diffuse/focal
Granulomas or lymphoid follicles?	Y/N
	If Y: infiltration into duct: Y/N
Interface hepatitis	Y/N
	If Y, zone 3 necrosis?: Y/N
	If Y, focal IH; mod IH (<50%); marked (>50%)
	If Y, cell type:
**Bile ducts**	
Normal number	Y/N
Epithelial injury	Y/N
	If Y: describe
Bile ductular proliferation/Ductular reaction	Y/N
Basement membrane thickening	Y/N

Concentric periductal sclerosis	Y/N
**Vascular alterations**	
Terminal hepatic vein	Y/N:<br>If Y: perivenul fibrosis; subintimal fibrosis/SHN; other:
Portal vein branches	Y/N<br>If Y: extruded; subintimal thickening; other:
	Periportal/paraportal shunt vessels present? Y/N
Hepatic arterty branches	Thickened or smudged media: Y/N
Sinusoids	Capillarization? Y/N
**PASD**	
Alpha-1 antitrypsin globules	Y/N
**Iron**	
Reticuloendothelial system (RES) iron grade	None, mild, moderate or above
If present, location	Hepatocyte; Sinusoidal lining cells; Both;<br>Gradient present: Y/N
**Reticulin**	
Abnormalities (eg hyperplasia, nodularity)	Y/N
**Copper stain**	
Granules	Positive/negative
**Comments**	
**Diagnosis**	
NAFL	Y/N
NASH	Y/N
Not NAFL	Y/N
Other	
